# Rapid Maxillary Expansion Treatment in Patients with Cleft Lip and Palate: A Survey on Clinical Experience in the European Cleft Centers

**DOI:** 10.3390/jcm12093159

**Published:** 2023-04-27

**Authors:** Nadiajda Khdairi, Talal Halilah, Mohannad Khandakji, Theodosia Bartzela

**Affiliations:** 1 Independent Researcher, 13086 Berlin, Germany; nadia_khd@yahoo.com (N.K.); talalhalilah86@gmail.com (T.H.); 2 Dental Department, Hamad Dental Center, Hamad Medical Cooperation, Doha P.O. Box 3050, Qatar; mohannad.khandakji@hotmail.com; 3 Department of Orthodontics and Dentofacial Orthopedics, Charité–Universitätsmedizin Berlin, Corporate Member of Freie Universität Berlin and Humboldt-Universität zu Berlin, 14197 Berlin, Germany; 4 Department of Orthodontics, Faculty of Medicine Carl Gustav Carus, Technische Universität Dresden, 01307 Dresden, Germany

**Keywords:** cleft lip and palate, rapid maxillary expansion, secondary alveolar bone graft, treatment protocols, survey, complication, European cleft centers, orthodontics

## Abstract

Cleft lip and palate patients require complex interdisciplinary treatment, including maxillary expansion and secondary alveolar bone grafting. However, the evidence on these treatment procedures and outcomes is lacking. Therefore, this study aimed to survey the subjective observations of European maxillofacial surgeons and orthodontists on the maxillary expansion and bone grafting treatment protocols and the associated complications. An online questionnaire was sent to 131 centers. The questions assessed the participants’ demographic data, maxillary expansion and alveolar bone grafting protocols, and the associated complications. Descriptive statistics and a *t*-test were used to analyze the data. The response rate was 40.5%. The average age for maxillary expansion was 9–10 years. The secondary alveolar bone grafting was planned 5–10 months after the expansion. The most common complications were asymmetric expansion, relapse, and fistula formation. The protocols and materials used vary widely among centers. Anatomical alterations and developmental processes, like tooth eruption adjacent to the cleft, should be seriously considered for treatment planning. This survey showed that there is still a lack of consensus on these treatment procedures. Further clinical trials should focus on long-term outcome evaluation to identify treatment components for optimal alveolar bone substitution and transversal maxillary expansion treatment in patients with clefts.

## 1. Introduction

Cleft lip and/or palate (CL/P) is the most common craniofacial malformation resulting from incomplete fusion of the facial prominences. The prevalence of CL/P varies according to sex, ethnicity, and geographic origin [1]. Approximately one in 700 children in Europe is born with a CL/P [1]. The etiology of cleft lip and palate (CLP) is multifactorial. Both genetic and environmental risk factors contribute to etiopathogenesis, but the exact mechanisms are not clearly defined [2,3,4].

Moreover, patients with more severe CLP phenotypes and isolated cleft palates are more likely to have associated congenital malformations and syndromes [2]. They often present with developmental and congenital dental anomalies in tooth number (agenesis, supernumerary teeth), shape, and position, in addition to severe malocclusions affecting the primary and permanent dentition [5,6,7]. Therefore, CLP patients require an early and complex interdisciplinary approach.

The surgical interventions required for CLP repair lead to scar tissue formation, palatal muscle strain, and constriction of the maxillary complex [8,9]. Furthermore, intrinsic factors affecting palatal growth may also contribute to maxillary constriction [10], often resulting in canine impaction [11]. Moreover, the lower tongue position stimulates mandibular growth, leading to crossbite and Class III malocclusion [5].

Maxillary expansion and secondary alveolar bone grafting (SABG) are essential for managing transverse skeletal discrepancy and alveolar integrity. The maxillary expansion increases the palatal volume, allows normal growth and development, and provides more space for proper tongue posture [12]. 

The SABG is a well-established procedure in the treatment of CLP patients. It stabilizes and provides continuity of the maxillary complex, facilitates tooth eruption, permits orthodontic tooth movement, and enhances facial appearance [13]. Both autologous and allogenic bone can be used for this procedure [14].

In non-cleft patients, rapid maxillary expansion (RME) is a well-documented treatment approach for maxillary transverse deficiency [15,16]. Various complications of RME using tooth-borne expanders in non-cleft patients have been described [17]. The most common complications are buccal tilting of the anchor teeth, buccal bone fenestration, and root resorption. Recently published data suggested that bone-borne or hybrid tooth-bone-borne RME might benefit both non-affected [18] and affected patients [19]. Hence, increased sutural opening and reduced tooth tipping were observed compared with conventional tooth-borne RME, but the evidence remains limited [18]. In patients with CLP, the mid-palatal suture resistance is less or negligible compared with non-cleft individuals. As a result, lighter expansion forces are required [20]. Therefore, special expansion protocols should be considered for patients with affected craniofacial structures [21,22,23]. 

In patients with CLP, the timing of RME compared with the SABG is fundamental for the expansion prognosis and the alveolar bone volume preservation. Many authors favor transverse orthodontic expansion before bone grafting. Advantages such as improved access when repairing the nasal floor, better postoperative healing and hygiene, reduced anatomical resistance, and a decreased probability of reopening the oronasal fistula have been reported [24]. However, recent data show that maxillary expansion after SABG is, at some point, advantageous [24,25] due to the applied dynamic load and the narrower defect to repair [24,26]. Functional stimuli, such as tooth eruption, orthodontic tooth movement, and expansion, could increase the success rate of the bone graft [26]. Nevertheless, expansion after bone grafting compromises the healing process and increases the incidence of fistula formation [27]. A literature review by Wang et al. [28] reveals that no randomized clinical trials provide evidence of the outcome and stability of palatal expansion in relation to SABG. 

Despite improving CLP care in Europe in the last two decades, there is a lack of evidence on RME timing and long-term treatment stability in patients with CLP [29]. This study was performed to give insight into the experience and subjective observations of the maxillofacial surgeons and orthodontists on the treatment protocols and outcomes of RME and SABG in patients with CLP in the European cleft centers. 

## 2. Materials and Methods

### 2.1. Study Design

This study was approved by the Ethics Committee of the Charité–Universitätsmedizin Berlin (EA4/085/19). The authors conducted an online cross-sectional European survey in the English language. An online survey platform (Survio.com) was used.

The questionnaire (Appendix A) consisted of 38 questions, requiring approximately 6–10 min to complete. It mainly consisted of multiple-choice questions, and multiple responses were possible. The questions assessed the following aspects:

- Participants’ background expertise and the center’s general information
- Protocol of RME (timing related to SABG, types of appliances used, activation and retention protocol)
- Generalized, technical, and oral complications

All participants received an email invitation with information on the research and a link to the online survey. The first email invitation was sent on 29 March 2020. A monthly reminder was sent to increase participation until October 2020. The questionnaire was available for response until 3 December 2020. The survey was voluntary, and the data was collected and processed anonymously.

### 2.2. Study Population

The questionnaire included orthodontic and surgical aspects. Therefore, orthodontists and/or craniofacial surgeons in European CLP centers were the target population. As both specialists on the cleft team possess the knowledge necessary to complete the questionnaire, only one participant was contacted from each center. Nevertheless, in case of difficulties, participants were encouraged to forward their survey link to another team member who could complete the survey to their best knowledge.

The EUROCLEFT project book [30], in combination with an online search for publicly available updated addresses, was used as a guide to extract the centers and their email accounts. In total, 150 European CLP centers were found. The questionnaire was distributed to 131 European CLP centers. We could not find the email addresses of the remaining 19 centers; therefore, we excluded them from this study.

### 2.3. Statistical Analysis

The data was managed and stored using Microsoft Excel^© 2023^. Statistical analysis was performed using the statistical software Stata/SETM (version 11.1). The results were analyzed using descriptive and analytic statistics, including means, frequencies, and standard deviations. A *t*-test was used to check for possible associations between variables. The significance level was set at *p* < 0.05.

## 3. Results

### 3.1. Participants

**Figure 1:**
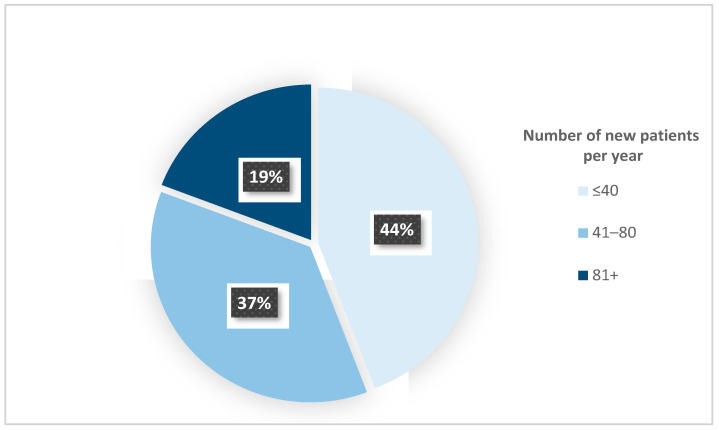
The annual centers’ caseload of patients with CLP.

In total, 54 questionnaires out of 131 were returned, corresponding to a response ratio of 40.5%. Most clinicians (70%) have at least ten years of treatment experience with CLP patients. In 44% of the centers, a caseload of fewer than 40 patients per year was reported. Only 19% of the participating centers have more than 80 CLP patients per year, and 37% have between 41 and 80 patients (Figure 1). Orthodontists comprised most participants (84%), and the remaining 16% were maxillofacial surgeons. More than half of the participants (68%) use RME to treat CLP patients.

### 3.2. RME Protocol and Relation to the SABG

**Figure 2:**
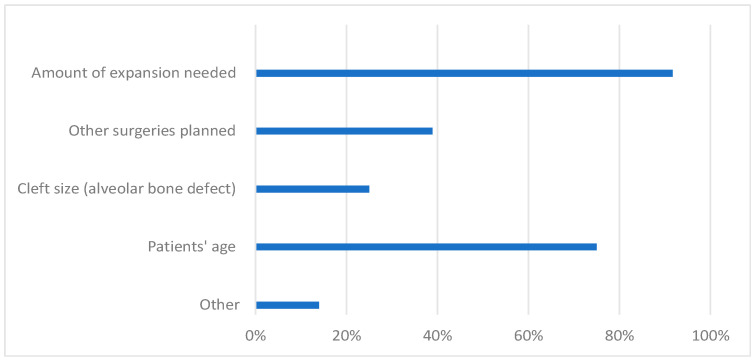
Factors affecting the timing of RME treatment according to the clinicians (percentage of the participants).

Factors affecting the timing of RME were mainly the patient’s age and the amount of expansion required. Some participants also considered other factors such as surgeries planned, cleft size, lateral incisor and/or canine root development, and the severity of the functional mandibular shift. In addition, ENT (Ear, Nose, Throat) problems, lack of space in the dental arch, logopedic indications, and patients’ cooperation were also considered (Figure 2). 

**Figure 3:**
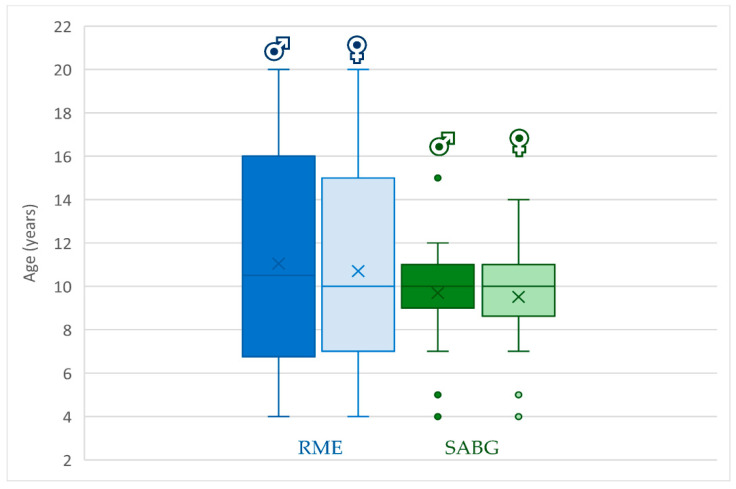
Age span for RME and SABG for males and females (Dots placed past the line edges indicate outliers).

The average age for performing RME was 9.4 ± 2.1 years in females and 9.9 ± 2.2 years in males. The youngest patients treated with RME were four years old, and the oldest were 20, both males and females. The average age for SABG was around 9.6 ± 1.8 years in females and 9.7 ± 1.8 years in males (Figure 3). 

**Figure 4:**
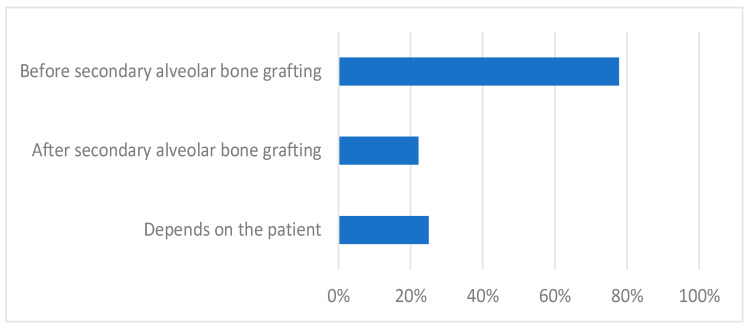
Timing of RME in relation to SABG according to the clinicians (percentage of participants).

The iliac crest was the bone of choice for the SABG for 82% of the responders. Other sources of bone grafts, such as the mandibular symphyseal bone, tibia, and allogenic tissue-engineered bone, were used by less than 10% of the participants. Clinicians were asked about the timing of RME in relation to SABG and were allowed to give multiple answers. The majority of the clinicians (78%) perform RME before SABG, while 22% perform RME after SABG. Since this question allowed for multiple answers per participant, 25% of clinicians also indicated that their choice of timing was patient-dependent (Figure 4). An estimated time between SABG and RME treatment provided by the cleft team experts ranged between a minimum of 5.5 ± 3.6 months and a maximum of 15.3 ± 10 months. Most responders (84%) performed SABG before the canine eruption, and only 2% performed it independently of the canine eruption stage. Almost 18% of the participants plan SABG, depending on the developmental stage of the lateral incisor. 

The average duration of the active RME phase was 1.8 ± 0.9 months; the shortest time was two weeks, and the most prolonged period was four months. For most participants (81%), the activation protocol was one to two turns daily.

### 3.3. RME Appliances and Retention Protocol

Tooth-borne expanders were used by 97% of the clinicians, and bone-tooth-borne hybrid expanders by 22%. Less than 20% of the responders used other devices, such as bone-borne and tooth-tissue-borne expanders. Most participants (77%) used the standard screw for the RME appliance. Fan expanders were used by 31%, and only a few participants used other screws like memory (5.7%) and super screws (5.7%).

For retention, most clinicians (83%) used the same RME appliance as in the active phase. Removable appliances, a quad-helix, or a transpalatal arch were used by 20% of the clinicians. The average retention period ranged from 8 ± 5.5 to 15 ± 9.5 months.

### 3.4. Treatment Complications

Several complications related to the appliance were reported. Loosening of the RME device was the most frequently reported technical complication, noticed in 1.6 ± 1.9 out of ten patients. Almost 1 ± 1.5 out of ten patients experienced complications such as screw blockage, appliance breakage, and confusion on how to activate the screw.

The most frequently reported general side effect in around 2 ± 2.3 patients out of ten was pain. On average, 1 ± 1.5 out of ten patients had headaches and dizziness. Less than one out of ten patients had other general complications such as metal allergies, a feeling of pressure or discomfort in the nose, difficulties in speaking, swallowing, and oral hygiene maintenance. 

**Figure 5:**
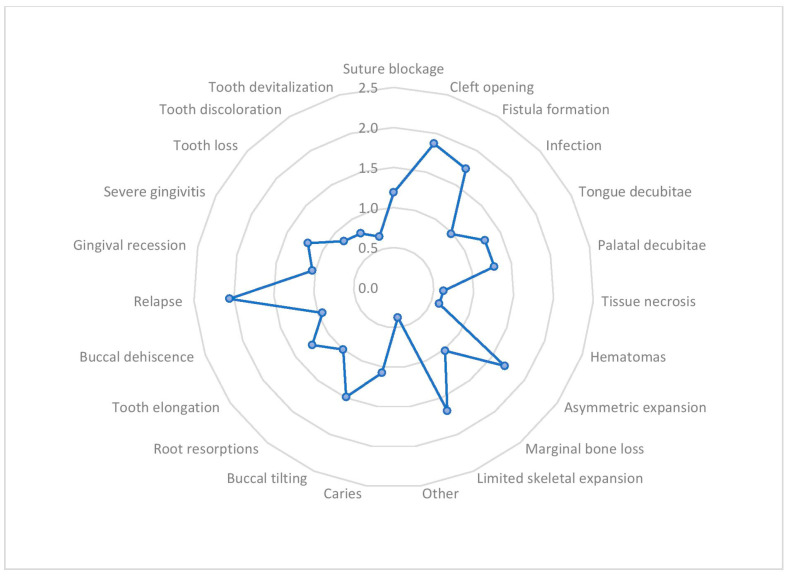
The average frequency of intraoral complications of RME in out of ten patients.

Relapse and instability were the most frequent intraoral complications. In around 2 ± 2.2 out of ten patients, cleft openings, fistulas, asymmetrical or limited skeletal expansion, and buccal tilting of anchor teeth were noticed. Furthermore, intraoral complications such as failure of maxillary suture opening, infection, decubitus on the tongue or the palatal mucosa, and tissue necrosis and/or hematomas were noticed in around one out of every ten patients. Other complications include buccal bone thickness and marginal bone level loss in the anchored teeth, elongation of non-anchor teeth, caries, root resorptions, severe gingivitis, and buccal dehiscence, which were observed in around one out of ten patients. Less frequently observed complications such as buccal fenestrations, gingival recession, tooth loss, discoloration, and devitalization of teeth were reported (Figure 5).

The nasal widening was seen in 1.5 ± 2 patients out of ten and was the most reported extraoral complication. Swelling, reddening, exostosis on the nasal bridge, and epistaxis were less frequently observed (less than one out of ten patients). 

In cases where the SABG was performed before RME, 24% of the clinicians noticed bone resorption in the SABG, 36% experienced no resorption, and 39% were unaware of it. Half of the participants reported up to 25% bone resorption when the SABG was performed before the RME. Most clinicians (70%) never observed resorption of more than 75% of the bone graft when SABG was performed before RME. The most common observed amount of bone resorption was 1–25% when SABG was performed after RME, according to 58% of the participants. Nevertheless, when SABG was performed after RME, resorption of more than 75% of the bone graft was observed in less than 26% of the patients, according to half of the responders (Table 1). Soft tissue scarring from previous surgeries was a limitation for RME, according to 43% of the clinicians, and 11% were unsure if there was any effect. 

The correlation between clinicians’ years of experience and the complications of the RME was negative for most of the complications and statistically not significant (*p* > 0.05). The relapse of the RME and the retention period were not statistically correlated (*r* < 0.1). Similarly, no correlation (*r* = −0.2, *p* = 0.3) was found between the clinicians’ experience and the amount of SABG resorption. The correlation between the average time between RME and SABG and the amount of SABG resorption was not significant (*p* = 0.4). There was a weak but statistically significant correlation between the amount of bone resorption and the relapse rate (*r* = 0.5, *p* = 0.02).

Most centers (89%) keep records of their patients treated with RME. More than 80% of the participants used dental models and photographs for patient documentation. Most clinicians (69%) used radiographs as part of their protocol. Some participants indicated using computed tomography (23%) and occlusal bite registration (34%). Only 6% used periodontal probing as part of their patient records.

## 4. Discussion

The interaction of different disciplines on a national or international level is demanded in managing patients with craniofacial anomalies [5,6]. Meticulous patient documentation, well-planned longitudinal studies, sharing clinical experience, and patient values integration would provide the best scientific evidence for treating patients with CLP. Healthcare models should focus on prevention, identifying risk factors, and population groups for integrated and long-term personalized care [2,3,4,31]. Furthermore, patients with CLP have broad phenotypic variability and different degrees of severity. In many instances, late identification or late expression of some phenotypes makes a precise diagnosis of an associated malformation or syndrome challenging [2,31].

Patients with CLP are more likely to have a missing midpalatal suture [32]; in addition, early surgical interventions can disrupt the intrinsic growth potential, resulting in maxillary hypoplasia [33]. Procedures like RME and SABG are necessary for CLP patients as they bear aesthetic and functional advantages. 

There is no consensus on the treatment protocols for patients with CLP among the European centers [34]. Moreover, the lack of data on the RME protocol in relation to the SABG and the associated complications [28] led us to this survey. Experienced clinicians from the European cleft centers gave their insight on this topic. 

RME was performed before SABG in most of the centers (78%). The mean age of maxillary expansion has been reported at 9.4 ± 2.1 and 9.9 ± 2.2 years for females and males, respectively. The amount of maxillary expansion and the patients’ age were the most critical factors for the timing of the maxillary expansion. Besides these factors, other planned surgeries, such as the cleft size, canine eruption, crossbite, and forced bite guidance, were also substantially considered. Performing RME in adults is controversial, but it has to be evaluated if this also applies to patients with CLP [35,36]. 

According to this survey, the SABG was planned around 9–10 years ago, before the canine eruption. The recommended timing for SABG is between 9 and 11 years [37]. The stage of development of the canine or lateral incisor on the cleft side should also be taken into consideration instead of the patient’s age [38,39]. SABG is often performed just before the canine eruption, but if the lateral incisor is present and erupting on the cleft side, SABG can be performed earlier [38,39]. Only three participants performed SABG around age five for quick normalized dental arch forms and periodontal support of the teeth adjacent to the cleft. An Early SABG procedure performed before or during the eruption of the lateral incisor could benefit the patient, but more studies are needed to explore this approach [40].

The SABG, after maxillary expansion, can stabilize the expanded maxilla and prevent relapse [8,41,42,43], which agrees with most of the responders to this survey. SABG can stabilize the expanded alveolar segments and allow the spontaneous eruption of these teeth in the grafted area [44]. However, relapse has also been described when using this protocol [45]. On the other hand, more recent literature describes the benefits of SABG before RME [15,46,47,48], claiming that this practice supports the mid-palatal suture opening and can provide more stability for RME. However, the results can be unpredictable [25]. In our study, only 22.2% of the clinicians performed SABG before RME.

There are different approaches when considering the period between the two procedures. Around 6–12 weeks after SABG, expansion can be initiated [43,49]. Others recommend waiting for at least three months after grafting [25]. Further research is needed to investigate the appropriate timing for RME after SABG [46]. The average period between SABG and RME was 5.5–15.3 months. The duration of this period was not statistically correlated with the amount of SABG resorption.

Maxillary alveolar reconstruction in patients with unilateral CLP was similarly effective using allogenic or autologous bone grafts [14]. Allogenic bone grafting using 3D plotting enhanced with osteogenic cells is a promising approach that requires further investigation [50]. Autologous bone grafts, both cortical or cancellous bones, can be used. Cancellous grafts incorporate faster than cortical bone, enabling tooth eruption [51]. Most of our participants used cancellous bone from the iliac crest, and 34% used the cortical bone. Other sources of bone grafts, such as the mandibular symphyseal bone, tibia, and allogenic tissue-engineered bone, were used by less than 10% of the participants. If a sizeable alveolar cleft is present, it can be reconstructed using bone from the iliac crest. In cases of smaller defects, bone substitutes from the chin can be used, as only a small amount can be collected [38]. The advantages of using mandibular bone are a shorter operative time and hospital stay, and no extraoral scar formation [52]. 

A review by Barillas et al. [53] stated that maxillary expansion after the SABG does not cause significant loss in bone volume or density. However, those authors [53] recommended caution with the reported results and suggested further experimental studies to evaluate the success of SABG in relation to RME. Some clinicians (24%) noticed bone resorption in the SABG independent of the timing of RME. It was reported that significant bone loss occurs between 3 and 12 months after grafting [46]. Placing the SABG under physiologic stress can help preserve the bone [13,54]. The most common amount of experienced bone resorption after/before RME was between 1 and 25%. In contrast, other researchers noticed that the average bone loss was 49.5% during the first year after surgery [55] but remained almost constant the following two years [55]. In some studies, the mean bone graft volume loss was around 36% [13,56]. 

Using tooth-borne expanders such as the Hyrax has the advantage of working on the short clinical crowns of the primary dentition and increasing twisting rigidity [57]. Our results showed that 97% of clinicians use tooth-borne expanders, which coincides with a recent European survey [29].

Researchers agree that the latest treatment time for RME is during puberty for non-cleft orthodontic patients [15,28,46,58]. In this survey, the oldest age indicated by the participants was 20 years of age, and the youngest was four years. A Belgian research group recommends correcting severe transverse maxillary constriction at a relatively young age through combined maxillary osteotomy and orthodontic expansion, due to the absence of a healthy midpalatal suture [59]. As a result, the need for a three-piece osteotomy at an adult age is reduced [59].

When considering the retention protocols, most clinicians follow the 8–15-month retention phase. Few clinicians had a one-month only and the most 24 months retention period reported. In contrast, published data shows that most clinicians follow a protocol with a three-month retention phase [17]. However, the six-month retention phase is also prevalent. Our results suggested no significant correlation between the retention period and the relapse of RME, implying that it is case-specific. Palatal strain, scar tissue, and hard and soft tissue deficiencies in that area contribute to deterioration and instability, making it more complex compared with non-cleft patients. 

The treatment complications in our questionnaire were divided into technical, general, intra- and extra-oral. The most common technical difficulty reported in the literature [17] was the loosening of the appliance, which corresponds with our results. Moving to the general complications, pain was the most common one. As other researchers have reported, the pain was mainly noticed in the molar region and was greater in patients who received the activation twice daily [60]. RME with an up-to-two-times-daily activation protocol was employed by most European centers [29]. Our results showed that most clinicians used one to two activations daily, and only two clinicians used them three to four times-a-day. Pain was the most common complication. 

Although most clinicians had more than ten years of experience, the most common intraoral complications prevailed. Relapse and instability were the most common intra-oral complications, in addition to fistula formation and asymmetric expansion. A statistically significant correlation was found between the amount of bone resorption and the relapse and instability rates (*r =* 0.5, *p* = 0.02). Buccal tilting up to 24° of the first molars is due to the teeth tilting and the deformation of the alveolar processes [61]. Figueiredo et al. concluded that buccal tiling was similar on both cleft and non-cleft sides, and symmetric expansion was achieved, but more effectively in the molar area [62]. Widening of the nose was the most common extraoral complication, according to our participants. Since nose growth takes a long time, the changes are discreet [17]. However, recent literature reveals that nasal and alar base widths increased significantly after expansion [63,64]. A nasal widening of around 2 mm was seen in a study [65] evaluated through photometric analysis. Epistaxis is a less common complication, mainly occurring after surgically assisted palatal expansion [66].

Almost all clinicians keep records of the CLP patients treated with RME, such as dental models and photographs. Computed tomography was used by 23%, similar to the results reported in recent literature [34,63]. Considering that CLP is a 3D facial deformity, using cone beam computed tomography helps achieve better care for those patients [67].

The sex distribution and phenotyping of the CLP patients were not clearly differentiated in this survey. Moreover, some associated malformations, whether isolated or as part of an underlined syndrome, are not diagnosed before the 4th or 5th year of age because of phenotypic variability or mild expression [68]. Some syndromes may be identified later in life because they are associated with learning difficulties or psychiatric symptoms, such as the 22q11DS syndrome [69]. Therefore, it would be hard for the participants to make a strict differentiation between syndromic and non-syndromic CLP individuals.

This survey might not represent all European centers, but almost half of the contacted centers (*n* = 54), providing a response rate of 40.5%, shared their long-standing experience in the field. Most of the responders had clinical experience of more than ten years, and 37% had more than 20 years of experience treating CLP patients, which adds to the strength of these findings. In addition, most of the responses correlated with the evidence-based literature. The CLP centers (*n* = 77) that did not respond to our questionnaire can be related to the tight schedules of clinicians [70]. Due to the anonymous data collection in this study, it is hard to rule out response bias. In the literature, there is no agreed-upon standard for acceptable response rates. However, low response rates increase the probability of response bias [71,72]. Our response rate of 40.5% is comparable to similar surveys in this field [30,34]. More studies are required globally to provide adequate evidence for treating these patients [73]. 

Further research, surveys, and randomized clinical trials should focus on the preferred bone for dentoalveolar reconstruction and the timing of RME in relation to SABG for the best bone volume preservation in treating patients with CLP.

## 5. Conclusions

The largest part of the responders used the following protocol:

- The average age for performing RME was 9–10 years. Taking into consideration the amount of expansion and the planned SABG.
- The SABG was planned 5–10 months after the RME.
- The canine root maturation stage and, in some cases, the lateral incisor developmental stage define the SABG timing.
- The retention period of RME was approximately 8–15 months after the last activation using the same orthodontic device.

Most participants have long-standing experience and a substantial caseload per year, which adds to the strength of these findings. Nevertheless, a lack of consensus exists on RME and SABG protocols, timing, and stability used for CLP treatment. 

## Figures and Tables

**Table 1:** Comparison of the reported bone resorption (%) when SABG is performed before or after RME.

	Percentage of Clinicians	
**SABG resorption**	SABG before RME (%)	SABG after RME (%)
yes	24.2	24.2
no	36.4	39.4
Do not know	39.4	36.4
**Bone resorption (%)**		
none	36.7	32.3
1–25	50	58.1
25–50	10	9.7
50–75	3.3	0
75–100	3.3	0
Patients (%) with substantial * **bone resorption**		
none	70	50
<26	26.7	50
26–50	3.3	0
51–75	0	0
>76	0	0

* Resorption of 75–100% of the grafted bone.

## Data Availability

The data presented in this study are available on request from the corresponding author.

## Appendix A

**Table A1:** A survey on complications of rapid maxillary expansion in cleft lip and palate patients in the European cleft centers.

What is the average CLP caseload per year in your center?	⚬ ≤40 ⚬ 41–80 ⚬ 81+
2. What is your specialty? Select one or more answers	⚬ Maxillofacial surgery ⚬ Oral surgery ⚬ Plastic Surgery ⚬ Pediatric surgery ⚬ ENT Surgery ⚬ Orthodontics ⚬ Maxillofacial surgery resident ⚬ Oral surgery resident ⚬ Plastic surgeon resident ⚬ Pediatric surgery resident ⚬ Orthodontic resident ⚬ Other, specify…………
3. How many years of experience treating CLP patients do you have?	⚬ 0–5 years ⚬ 6–15 years ⚬ 16–25 years ⚬ 26+ years
4. Do you use RME for the treatment of cleft patients?	⚬ Yes ⚬ No
5. Approximately how many CLP cases did you treat with RME last year?	⚬ ≤40 ⚬ 41–80 ⚬ 81+
6. Which of the following factors affect your timing choice to perform the RME? Select one or more answers	⚬ Patient age ⚬ Cleft size ⚬ Amount of expansion needed ⚬ Other surgeries planned ⚬ Others, specify
7. At what age, on average, do you perform RME in patients with a cleft?	Average age for boys………………….. Average age for girls…………………..
8. What is the age span (maximum and minimum age) in which you perform the RME for cleft patients?	Age span for boys; minimum at…..maximum at……. Age span for girls; minimum at…..maximum at…….
9. Approximately at what age do you perform bone grafting in the cleft region?	Average age for boys………………….. Average age for girls…………………..
10. What type of bone graft do you use? Select one or more answers	⚬ Iliac crest (Cancellous bone) ⚬ Iliac crest (Cortical bone) ⚬ Calvarium ⚬ Mandible ⚬ Tibia ⚬ Rib ⚬ Allogenic bone ⚬ Tissue engineered bone ⚬ Other, specify………….
11. When do you perform RME in relation to the secondary alveolar bone graft? select one or more answers	⚬ Before secondary alveolar bone grafting ⚬ After secondary alveolar bone grafting ⚬ Depends on the case, specify: ……………….
12. Please specify the time span between the bone grafting and the RME treatment in days/ weeks/or months?	Minimum……………….. Maximum……………….
13. When do you perform the secondary alveolar bone graft in relation to the permanent maxillary canine eruption?	⚬ Just before the canine eruption ⚬ Just after the canine eruption ⚬ Not related to the canine eruption ⚬ Other, please, specify ……………..
14. What is the usual duration of the active RME phase in weeks/months?	………………………….
15. What is the frequency of daily activation?	⚬ 1–2 times ⚬ 3–4 times ⚬ Other, specify ……………
16. What type/types of RME appliances do you use?	⚬ Tooth-borne expanders (e.g., Hyrax, Issacson) ⚬ Bone-borne expanders, anchored with mini implants ⚬ Bone-tooth-borne hybrid expanders ⚬ Tooth-tissue borne expanders (e.g., Haas, Derichsweiler, Arnold, IPC expander, Bonded expander) ⚬ Other, specify: ………….
17. What type of screw do you usually use? Select one or more answers	⚬ Standard ⚬ Memory ⚬ Fan expander ⚬ Mini Palex ⚬ Click screw ⚬ Super screw ⚬ VECS screw ⚬ Other, specify: ……….
18. What type of retention did you use after the RME?	⚬ The same passive RME appliance ⚬ Upper removable appliance ⚬ Other, specify: …………
19. What is the maximum and minimum duration of the retention period after RME in weeks/ months/ years?	Minimum ……………………….. Maximum ………………………..
20. What type of technical complications do you face when using RME? (Please, indicate the frequency of the complication per 10 patients)	⚬ Loosening of RME device: …… ⚬ Screw blockage:….. ⚬ Breakage:…… ⚬ Other, specify: ………..
21. If you have noticed complications besides the ones mentioned, please specify here	………..
22. Which of those general complications do you face when using the RME? (Please, indicate the frequency of the complication per 10 patients)	⚬ Pain: ……… ⚬ Headache: ……… ⚬ Dizziness: …….. ⚬ Other, please specify: ………
23. If you have noticed complications besides the ones mentioned, please specify here	…………..
24. Which of the listed intra-oral complication do you face when using the RME? (Please, indicate the frequency of the complication per 10 patients)	⚬ Suture not opening: ………….. ⚬ Root resorptions: ………….. ⚬ Buccal tilting of the anchor teeth:……………… ⚬ Elongation of non-anchor teeth: ……………….. ⚬ Loss of marginal bone level in anchored teeth: ……………. ⚬ Loss of buccal bone thickness: …………………. ⚬ Gingival recession: ……………… ⚬ Opening of the cleft: ……………… ⚬ Fistula: ………………. ⚬ Decubitae (pressure ulcers) on the tongue: …………… ⚬ Decubitae (pressure ulcers) on the mucosa: ………… ⚬ Severe gingivitis: ………………… ⚬ Buccal dehiscence: ………………….. ⚬ Buccal fenestrations:………………. ⚬ Limited skeletal expansion: ………………. ⚬ Tooth loss:……………….. ⚬ Hematomas:…………….. ⚬ Relapse and instability of the expansion:…………… ⚬ Tissue necrosis:…………. ⚬ Tooth discoloration:…………… ⚬ Infection:……………. ⚬ Devitalization of teeth:…………. ⚬ Asymmetric expansion:…………….. ⚬ Caries:………… ⚬ Other, specify:…………..
25. If you have noticed complications besides the ones mentioned, please specify here	………..
26. Which of the following extra-oral complication of RME did you face? (Please, indicate the frequency of the complication per 10 patients)	⚬ Swelling:…..… ⚬ Reddening:…….. ⚬ Nasal widening:………. ⚬ Exostosis on the nasal bridge:……… ⚬ Epistaxis:………… ⚬ Other, specify:………
27. If you have noticed complications besides the ones mentioned, please specify here	……………..
28. Did you notice bone resorption in the secondary alveolar bone graft before RME?	⚬ Yes ⚬ No ⚬ I do not know
29. What is the most common amount of bone resorption you see in the cleft area graft before the RME?	⚬ None ⚬ 1–25% of bone resorption. ⚬ 25–50% of bone resorption. ⚬ 50–75% of bone resorption ⚬ 75–100% of bone resorption with no continuous bony bridge through the cleft
30. What’s the percentage of the cases that had 75–100% of bone resorption before RME? (75%–100% of bone resorption with no continuous bony bridge through the cleft)	⚬ None ⚬ 1–25% ⚬ 26–50% ⚬ 51–75% ⚬ More than 76%
31. In case the RME is done after the bone grafting, did you notice bone resorption in the secondary alveolar bone graft?	⚬ Yes ⚬ No ⚬ I do not know
32. What is the most common amount of bone resorption you see in the cleft area after RME?	⚬ None ⚬ 1–25% of bone resorption. ⚬ 25–50% of bone resorption. ⚬ 50–75% of bone resorption ⚬ 75–100% of bone resorption with no continuous bony bridge through the cleft
33. What’s the percentage of the cases that had 75–100% of bone resorption after RME (75–100% of bone resorption with no continuous bony bridge through the cleft)?	⚬ None ⚬ 1–25% ⚬ 26–50% ⚬ 51–75% ⚬ More than 76%
34. Have you noticed a limitation for the maxillary expansion due to soft tissue scarring from previous surgeries?	⚬ Yes ⚬ No ⚬ Some time ⚬ I do not know
35. Do you document and keep records of your patients treated with RME?	⚬ Yes ⚬ No
36. What type of documentation do you use?	⚬ Dental models ⚬ Photographs ⚬ Radiograph ⚬ Occlusal bite registration ⚬ Periodontal probing ⚬ Computed tomography ⚬ Others specify: ………………….
37. Are you willing to grant us access to those documents for a limited period of time, if needed?	⚬ Yes ⚬ No
38. If you answered the previous questions with yes, please provide your contact details below.	……………..
